# Immunohistochemistry and alternative FISH testing in breast cancer with *HER2* equivocal amplification

**DOI:** 10.1007/s10549-018-4755-5

**Published:** 2018-03-22

**Authors:** Sally Agersborg, Christopher Mixon, Thanh Nguyen, Sramila Aithal, Sucha Sudarsanam, Forrest Blocker, Lawrence Weiss, Robert Gasparini, Shiping Jiang, Wayne Chen, Gregory Hess, Maher Albitar

**Affiliations:** 1 NeoGenomics Laboratories, Research and Development, 31 Columbia, Aliso Viejo, CA 92656 USA; 2 Cancer Treatment Centers of America, Philadelphia, PA USA; 3 Symphony Health, Conshohocken, PA USA

**Keywords:** HER2, Equivocal, FISH, IHC, Breast cancer

## Abstract

**Purpose:**

While *HER2* testing is well established in directing appropriate treatment for breast cancer, a small percentage of cases show equivocal results by immunohistochemistry (IHC) and fluorescence in situ hybridization (FISH). Alternative probes may be used in equivocal cases. We present a single community-based institution’s experience in further evaluating these cases.

**Patients and methods:**

Between 2014 and 2016, 4255 samples were submitted for *HER2* amplification testing by alternative probes, *TP53*, *RAI1*, and *RARA*. Of the patients tested by FISH, 505/3908 (12.9%) also had IHC data.

**Results:**

Most (73.9%) FISH equivocal cases remained equivocal after IHC testing. However, 50.5% of equivocal cases were classified as *HER2* amplified by alternative probes. Most cases were positive by more than one probe: 78% of positive cases by *RAI1* and 73.9% by *TP53*. There was a significant difference between IHC and FISH alternative testing (*p* < 0.0001) among the equivocal cases by conventional FISH testing, 44% of IHC negative cases became positive while 36% of the positive IHC cases became negative by alternative FISH testing. Available data showed that 41% of patients were treated with palbociclib and were positive by alternative FISH.

**Conclusion:**

The prevalence of double *HER2* equivocal cases and the discrepancy between IHC and alternative FISH testing suggest that FISH alternative testing using both *RAI1* and *TP53* probes is necessary for conclusive classification. Because almost half of FISH equivocal cases converted to *HER2* amplified upon alternative testing, clinical studies to determine the benefit of anti-HER2 therapy in these patients are urgently needed.

## Introduction

Testing human epidermal growth factor receptor 2 gene amplification (*HER2; ERBB2*) is important in breast cancer (BC) and other cancers. *HER2*, on the long arm of chromosome 17, is amplified and its encoded protein is overexpressed in 10–25% of BCs [1]. Without anti-HER2 treatment, *HER2*-amplified tumors are associated with poor prognosis [1, 2], but when treated with therapies targeting HER2, such as trastuzumab, pertuzumab, and TDM-1, they are shown to markedly improve survival [3–5]. Moreover, these patients can be excluded from treatment for which *HER2*-amplified tumors have been shown to be resistant, such as anthracyclines, taxanes, cyclophosphamides, and tamoxifen [6]. Anti-HER2 therapy is expensive and cardiotoxic in some patients, and *HER2*-unamplified patients should not be prescribed HER2 inhibitors [4, 7, 8].

*HER2* amplification testing determines if a patient is eligible to be treated with anti-HER2 therapy [9–11]. *HER2* amplification testing is performed either using immunohistochemical analysis (IHC), which tests for protein expression, or fluorescence in situ hybridization (FISH), which tests for gene amplification [2]. Many laboratories use IHC analysis as a primary assay, with “reflex” FISH testing for IHC equivocal results. In 2013, the American Society of Clinical Oncology and the College of American Pathologists (ASCO–CAP) updated the *HER2* interpretative guidelines and revised the equivocal category for *HER2* ISH, proposing a reflex test for such cases on the same specimen, with a different method or with an alternate ISH chromosome probe [12]. Based on the 2013 ASCO–CAP guidelines, a sample is classified as equivocal by FISH when *HER2*:*CEP17* (centromere) is < 2.0, but *HER2* signals are  ≥ 4 but  < 6 [12]. The current ASCO–CAP guidelines increased the number of equivocal cases significantly from the previous 2007 one, so that 10–17% of tested BCs are estimated to be classified as equivocal by the new ASCO–CAP FISH testing guidelines [13–20].

A probe at or near centromere 17 has traditionally been used as a reference probe, because it presumably reflects the copy number of chromosome 17 [21]. However, the centromere probe region can also be amplified as a result of local or regional amplification [22–25]. Moreover, *HER2*-amplified cancers have complex genetic abnormalities and the *HER2* amplicon at 17q12 contains multiple genes, which may co-amplify with *HER2* [26]. The use of alternative chromosome 17 probes, including *RAI1* (retinoic acid induced 1; previously known as *SMS*), *RARA* (retinoic acid receptor), and *TP53* (tumor protein 53), allows an alternative calculation of the *HER2*:chromosome 17 ratio, providing an alternative FISH ratio score in cases with a complex *CEP17* pattern, including polysomy 17 [12, 21, 25]. The 2013 ASCO–CAP guidelines do not specify which alternative probes should be used or whether guidelines for FDA–approved probes can be extrapolated to alternate probes [12]. Alternative chromosome 17 probes currently used in these cases are *RARA*, *TP53*, and *RAI1*. These loci are distal from *HER2* on the long and short arms of chromosome 17 and in principle are presumed to be less influenced by *HER2* amplification [21]. In this paper, we report one reference laboratory’s experience in testing HER2 equivocal cases referred from various institutions.

## Methods

### Patient samples and methodology

Between late 2014 and late 2016, 4255 consecutive formalin-fixed paraffin-embedded (FFPE) BC samples were submitted for testing at NeoGenomics Laboratories (Aliso Viejo, CA) by alternative probes for *HER2* amplification due to equivocal results by conventional methods. The list of patients tested with alternative *HER2* probes was processed to generate HIPAA-compliant synthetic identifiers, subsequently submitted to Symphony Health (Conshohocken, PA) to obtain clinical information, including drug exposure [27]. By utilizing the same de-identification algorithm at Symphony, all patient-level records were matched and linked across setters of care. Symphony Health Solutions’ data warehouse contains longitudinal patient (220 million) data sources that capture adjudicated prescription (10 billion), medical, and hospital claims across the United States. This study was approved by the Western Institutional Review Board, Olympia, WA.

### Immunohistochemistry testing

From each tissue block, 4 µm sections were cut, deparaffinized in xylene, and dehydrated through alcohol changes. IHC for HER2 was performed with one of the FDA-approved assay kits (Lica Biosystem), according to standard methods as previously published and scored according to the 2013 ASCO–CAP Guidelines [28].

### Fluorescence in situ hybridization testing

Three probe kits were used for FISH analysis: (1) the US Food and Drug Administration (FDA)–approved *HER2* IQFISH pharmDx (Agilent, Santa Clara, California). *HER2* equivocal panel contained *RARA* (17q21.2), *RAI1* (17p11.2), *TP53* (17p13.1), and *CEP17* (SHGC133091-RH42746) (Agilent, Santa Clara, California) for the *HER2* alternative panel. This *CEP17* probe is 436 kB, paracentromeric, and located at 17p11.2, while the centromeric probe used in conventional HER2 testing (*CEN17*) is located between 17p11.1–q11.1.

For each specimen, 4 µm sections were cut, deparaffinized, rehydrated, and heated in pretreatment solution [2-(N-morpholino) ethanesulphonic acid] at 97 °C for 10 m and then soaked in Tris/HCl buffer at room temperature (RT) for 3 m, twice. Then, the specimens were digested by proteinase K (25 mg/mL) at 37 °C for 45 m and agitated in Tris/HCl buffer at RT for 3 m, twice. Slides were then dehydrated in ethanol series at RT and air-dried. Ten µl of probe was applied to each slide, and the hybridization area was coverslipped. Co-denaturation of probe and specimen was performed at 66 °C for 10 m, and the slides were incubated at 45 °C for 90 m. Slides were washed in 2 × SSC/0.3% NP-40 at 63 °C for 10 m, and 10 µl of DAPI (4′6′- diamidino-2-phenylindole dihydrochloride) counterstain was applied before coverslipping. Slides were analyzed on a Zeiss Imager. Z2 fluorescent microscope using Isis FISH imaging systems (MetaSystems, Altlussheim, Germany) software. For each slide, fluorescent signals were counted by two independent observers in 40 cells (ASCO–CAP guidelines recommend a minimum of 20 cells per slide) [28]. Equivocal status for *HER2* was determined by the ratio of *HER2*:*CEP17* < 2 with an average *HER2* copy number ≥ 4.0 but < 6.0. Alternative testing was considered positive if the ratio using one of the alternative probes was ≥ 2.0. *TP53* scoring was not considered when the *TP53* signals were < 1.7 because the low number of signals in these cases is consistent with *TP53* 17p deletion rendering the probe an invalid control for ratio determination in these cases. In addition, a normalization approach was used as follows: *HER2* x *CEP17*/*TP53* x *CEP17*.

### Statistical analyses

Counts and frequencies were reported for each pre-specified measure and endpoint. The level of significance was defined as *α* = 0.05. Standard statistical tests were used to evaluate the correlations between variables including the Wilcoxon Matched Pairs Test.

## Results

### Patient characteristics

Of the tested 4255 FISH equivocal samples, 282 were duplicate samples either due to an inadequate first sample or due to testing of a second paraffin block from the same tumor. Therefore, there were 3973 unique patient samples. Of these, 65 had either inadequate tumor for evaluation (QNS) or inadequate number cells counted for conclusive results, yielding 3908 unique samples with FISH data (Table 1). Of the 3973 total unique patient samples, 97.0% (*N *= 3854) were matched to one or more claims data. The patient demographic profile of the study is 58% Caucasian, 10% African American, 4% Hispanic, and other or missing data in 28%.

**Table 1:** FFPE samples available for IHC and FISH alternative testing

FISH alternative testing		IHC (all cases)		
FISH result	Cases (*N*)	IHC score	Cases (*N*)	%
Duplicate	282			
QNS	65	0/1	137	24.6
Positive	1973	3	13	2.3
Negative	1935	2	406	73.0
Total	4255		556	100.0

*QNS* inadequate tumor for evaluation

### IHC in HER2 FISH equivocal cases

Of the 3908 patients tested by FISH, 556 (14.2%) also had IHC data. These data are summarized in Table 1. Of the 556 FISH equivocal cases, IHC was negative (score = 0/1+) in 137/556 (24.6%), and positive (score = 3+) in 13/556 (2.3%), and the rest, 406/556 (73.9%), were equivocal (double equivocal) by conventional testing.

### Duplicate testing by IHC

Patients tested by IHC included 96 cases tested twice using a different paraffin block from the same tumor. In the first test, 4/96 (4.2%) demonstrated an IHC score of 0, 32/96 (33.3%) a score of 1+ , 57/96 (59.4%) a score of 2+ , and 3/96 (3.1%) a score of 3+ . In the second test, 6/96 (6.3%) scored 0, 39/96 (40.6%) scored 1+ , 47/96 (49.0%) scored 2+ , and 4/96 (4.2%) scored 3. These results are shown in Table 2. There was a significant difference (*p* = 0.001) in the groups classified as 2+ and 1+ by first IHC: 44% of cases scored as 1+ on the first IHC test became equivocal (score 2+) on the second block test, and 40% of the equivocal cases on the first block test scored as negative and 7% as positive on the second block test (Table 2).

**Table 2:** Duplicate testing by IHC using a different paraffin block of the same tumor

First IHC score	No	Second IHC score	No	%	*p* value
0	4	0	1	25.0	NA
		1	3	75.0	
1	32	0	2	6.3	*p *= 0.001
		1	16	50.0	
		2	14	43.8	
2	57	0	3	5.3	*p* = 0.001
		1	20	35.1	
		2	30	52.6	
		3	4	7.0	
3	3	2	3	100.0	NA

### Discrepancy between IHC and alternative fish testing

After duplicates were excluded, 507 FISH equivocal cases had complete IHC and alterative FISH testing. As shown in Table 3, of the 121 negative cases by IHC (score 0 and 1+), 43.8% (53/121) became positive by alternative FISH. Although the number is small, 36.4% (4/11) of the positive cases by IHC became negative by alterative FISH testing. Of the equivocal cases by IHC (score 3+), 52.3% became positive. This was statistically significant (*p* < 0.0001). The high discordance in these cases as compared with average BC cases, most likely, reflects that the *HER2* equivocal cases by FISH are different with borderline amplification.

**Table 3:** Discrepancy between IHC and alternative FISH testing

Alternative FISH	IHC score 0 Negative		IHC score 1 Negative		IHC score 2 Equivocal		IHC score 3 Positive	
	No	%	No	%	No	%	No	%
Negative	14	63.6	54	54.5	178	47.7	4	36.4
Positive	8	36.4	45	45.5	195	52.3	7	63.6
Total	22	100.0	99	100.0	373	100.0	11	100.0

### Duplicate testing by FISH in HER2 equivocal cases

Of the cases tested by alternative FISH testing, 200 were tested twice using a different paraffin block. There was a significant difference in results between the first and the second test (*p *< 0.00001) when cases with complete results are considered. Some of the repeat testing was due to inadequate tumor for evaluation or indeterminate results due to availability of less than 50 cells for counting. If these cases are excluded, 18 of 97 negative cases (18.6%) became positive on second block testing and 16 of 82 of positive cases (19.5%) became negative on second block testing. The reason for this difference does not seem to be related to significant differences in the results of the same probes used in the first block test as compared with the second block test (Table 4). There was significant difference in results between different probes as well as between the two control probes used in the *HER2* testing: *CEN17* and the paracentromeric probe used with the *TP53* testing (*CEP17*). The difference in positive versus negative results is most likely due to minor difference in signals that may reflect heterogeneity within the tumor.

**Table 4:** Comparison between probes results in duplicate testing

Pair of variables		*Z*	*p* value
Measured probe	Reference probe		
*RAI1*	*TP53*	2.01977	0.04
*RAI1*	*RARA*	6.87694	< 0.00001
*RAI1*	*RAI1*	0.53653	0.59
*RARA*	*TP53*	8.168	< 0.00001
*RARA*	*RARA*	0.09232	0.93
*TP53*	*TP53*	0.49738	0.62
*TP53*	*RARA*	8.27438	< 0.00001
*CEP17*	*CEP17*	0.57489	0.57
*HER2*	*HER2*	0.73485	0.46
*CEP17*	*D17Z1*	5.59557	< 0.00001

### Alternative testing results

**Fig. 1:**
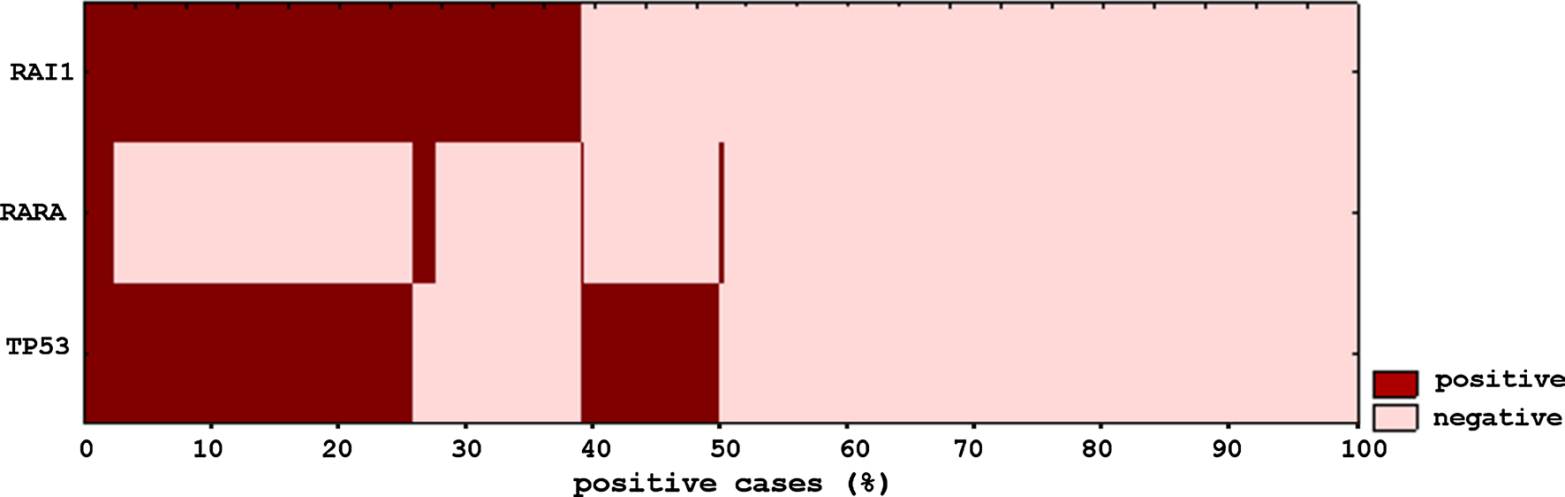
Representation of the overlap in results of the three probes used in classifying the tested equivocal cases. 38% of all cases were deemed positive based on *RAI1* probe, 36% based on the *TP53* probe, and 5% based on *RARA*

After excluding duplicates and cases without complete results, 1973 cases (50.5%)were reported positive by alternative *HER2* FISH testing, and 1935 cases (49.5%) were reported as negative (see Table 1). There was no significant difference in age (*p* = 0.06) between the two groups. However, there was significant (*p* < 0.0001) difference in the scores between the three probes (*RAI1*, *TP53*, and *RARA*). As shown in Fig. 1, thirty-eight percent of all cases (78% of positive cases) were deemed positive based on the *RAI1* probe, 36% of all cases (73% of positive cases) were considered positive based on the *TP53* probe, but only 30% of these cases were uniquely positive in *TP53* (11% of all cases and 22% of positive cases). This agrees with a previous study showing that *RAI*-identified FISH equivocal cases as amplified in almost 40% of all cases when used as the only alternative probe [12]. Only 5% of all cases (9% of positive cases) were considered positive based on *RARA*. However, if we exclude cases with *RAII* signals < 1.7, in a fashion similar to our approach to *TP53* probe, 10% of positive cases by *RAII* would be excluded, but half, 5%, of these cases would have been considered amplified by *TP53* probe.

Furthermore, it has been suggested that to account for variations in signal counts between tissue sections, the *HER2*:*TP53* ratio can be normalized based on the centromere counts in the two tissue sections and can be calculated as

$$\frac{HER2x[CEP17 of TP53]}{TP53x[CEN17 of HER2]}.$$

18.5 of total cases were positive by normalization (excluding any case with *TP53* signal < 1.7). Using this approach increases the positive cases to 54.2% of all equivocal cases. However, only 6% of the positive cases would be called positive based solely on normalization. Since there is a significant difference between the two centromere probes used, we do not think this normalization is appropriate and these cases are not reported as positive for *HER2* amplification in this study.

### Estrogen/progesterone (ER/PR) receptors status in HER2 equivocal cases

ER/PR evaluation using IHC was available on 225 samples that were classified as equivocal for HER2 by conventional testing. Of these 80 (35.5%) were reclassified as positive, 86 (38.2%) were reclassified as negative, and 59 (26.2%) remained as equivocal after alternative testing (Table 5). Significantly, more positive cases for HER2 by alternative testing were ER+/PR+ as compared with HER2 negative by alternative testing: 82% versus 59% (*p* = 0.0009). This is consistent with the expected higher hormone receptors positivity in cases with HER2 positivity and supports that alternative HER2 testing provides data in line with the conventional testing [29]. In contrast, cases remained equivocal for HER2 testing by alternative testing, which showed no significant difference in ER/PR positivity from the HER2 positive, 75% versus 82% (*p *= 0.15) or negative, 75% versus 59% (*p *= 0.14).

**Table 5:** Correlation between HER2 and HR status

	No	ER+		PR+		ER +/PR+	
HER2+	80	71	88.8%	66	82.5%	66	82.5%
HER2-	86	62	72.1%	54	62.8%	51	59.3%
HER2 equivocal	59	52	88.1%	46	78.0%	44	74.6%

### Therapy data in patients with equivocal fish testing

**Fig. 2:**
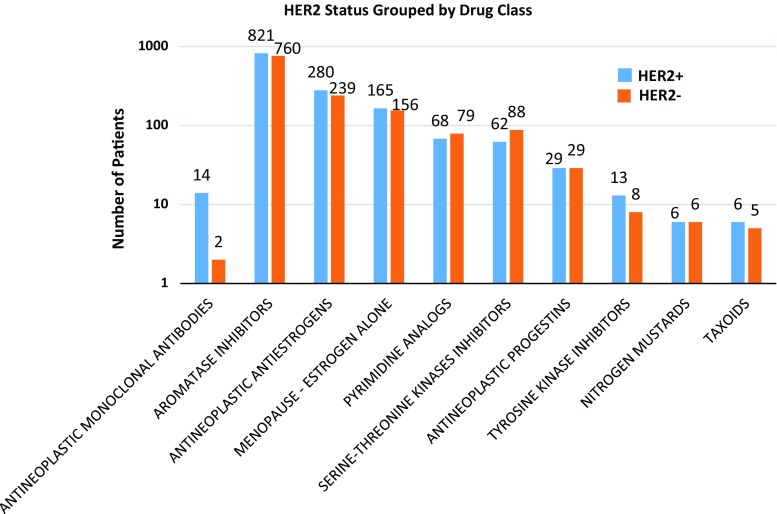
Except for treatment with anti-HER2, there was no significant difference in overall therapy between HER2-positive and HER2-negative cases as assessed by alternative FISH testing. Drugs are grouped into classes as shown

Although data may not complete, based on the information available in the claims data, few of these patients (*N *= 16) were treated with *HER2* inhibitors, while 124 patients were treated with palbociclib therapy, which is recommended for HER2-negative tumors. Therefore, proportionally few patients were treated according to the results of the alternative FISH testing, despite the availability of the results of the testing. However, this may not be accurate because of the pattern of dispensing anti-HER2 therapy in a hospital-based setting and some of these data may be absent from the dataset. Nevertheless, of the 16 patients treated with anti-HER2 therapy, two were negative by alternative FISH testing and 14 were positive. Pertuzumab was used in six of the trastuzumab-treated patients, five of whom were *HER2* positive by alternative testing. Of the 124 patients treated with palbociclib, 51 (41%) were positive for *HER2* by alternative FISH testing. To explore if there was an overall difference in the prescribed medications between cases classified as *HER2* positive and negative by alternative FISH testing, we grouped the therapy into the following categories: aromatase inhibitors, antineoplastic anti-estrogens, menopause—estrogen alone, pyrimidine analogs, serine–threonine kinase inhibitors, antineoplastic progestins, antineoplastic monoclonal antibodies, tyrosine kinase inhibitors, nitrogen mustards, and taxoids. There was no significant difference in therapy when drugs were grouped into these classes between positive and negative *HER2* cases by alternative testing (Fig. 2). There were 34 patients treated with everolimus. Of these, 14 were classified as *HER2* unamplified and 20 as *HER2* amplified.

## Discussion

While the overall concordance between FISH and IHC is high (94.1–97.6% in average BC cases), and a definitive result by IHC will be largely consistent with FISH, equivocal cases differ substantially [30–33]. Studies have shown that 20–28% IHC equivocal cases have gene amplification by FISH [20, 28, 30, 34]. The 2013 ASCO–CAP criteria for determining *HER2* amplification by FISH increase the number of equivocal cases significantly from the earlier 2007 criteria [21]. Classifying and understanding the biological and clinical behavior of equivocal cases is more difficult and requires more carefully designed biomarkers. Alternative FISH probes have been recommended for equivocal cases, but the reliability of such testing and the clinical relevance of the results are not well established. In the current study, we attempted to explore the relationship between IHC and FISH alternative testing in a large number of community-based samples that were referred to one reference laboratory and tested in relatively similar fashion. We also attempted to explore trends for treating this group of patients in community-based oncology practice.

Based on our data, 73.9% of FISH equivocal cases remained equivocal after IHC testing. Therefore, relying on IHC for resolving FISH equivocal cases is not adequate and most cases need to be resolved by other means. Furthermore, the poor reproducibility of IHC in HER2 testing [35, 36] and the significant discrepancy observed upon repeated testing of samples on a different block, in the same laboratory, using the same conditions, and interpreted by the same pathologists, confirm that relying on IHC in these cases may not be an acceptable approach. Our alternative FISH testing resulted in 52% of the equivocal cases becoming positive for *HER2* amplification. Furthermore, there was significant (*p *< 0.0001) difference between IHC testing and FISH alternative testing. This discrepancy most likely reflects that equivocal cases are different from average BC cases in terms of detecting *HER2* amplification. In fact, the difference in results when different blocks were tested by alternative FISH probes also suggests that these cases are different from average cases. In general, FISH testing is more reproducible and objective. The reproducibility seen in duplicate IHC testing is significantly lower (44% of IHC negative became equivocal and 40% of IHC equivocal became negative) than FISH (19% of alternative FISH positive cases became negative and 20% became positive on duplicate alternative FISH testing). There was a significant (*p *< 0.0001) difference in the scores obtained by each of the three probes (*RAI1*, *TP53*, and *RARA*), suggesting that they are not redundant. The *RAI1* probe was used for final classification of 78% of the positive cases. The *TP53* probe as a single marker for amplification was used in only 22% of cases. *RARA* was the single marker for amplification in only 5% of positive cases, which suggests that it has limited discriminatory value.

Clearly, FISH alternative testing provides valuable information and is relatively more precise than IHC in classifying *HER2* amplification. The demonstration that there is a significantly higher percentage of patients positive for ER+/PR+ in the cases that converted to HER2 positive by alternative testing also supports the value of alternative testing by FISH. Although a large percentage of equivocal cases become amplified, making them eligible for HER2-targeted therapies, there are no data available to determine whether this subgroup of patients will benefit. There is a clear necessity to evaluate response in patients identified as amplified by alternate probe in a clinical trial such as NCT01275677. In principle, approximately half of the patients in this study should have been considered for targeted anti-HER2 therapy. Although accurate and complete data on therapy in these community-based patients are not available, based on the available information, few of these eligible patients (*N *= 15) were treated with HER2 inhibitors. In contrast, 41% of patients who received a therapy that is indicated in *HER2*-negative patients were *HER2* positive by alternative FISH testing. Despite the concern over incompleteness of the data, the difference in number of patients treated with anti-HER2 therapy versus those who were treated as if *HER2* was not amplified, which suggests that in community-based practice, a high percentage of patients with *HER2* amplification are not being treated with HER2 inhibitors.

## Conclusion

In this study, we demonstrated that the majority of BCs with equivocal *HER2* FISH results remain equivocal after IHC testing. Alternative FISH testing of *HER2* amplification in equivocal cases provides a standardized approach resulting in classifying almost half the cases as amplified. While currently there are no conclusive data to support that these patients will respond to anti-HER2 therapy and clinical trials are needed to specifically answer this question, patients classified as *HER2* equivocal should be tested by alternative probes and should be considered for anti-HER2 therapy when positive.
